# CG Demethylation Leads to Sequence Mutations in an Anther Culture of Barley Due to the Presence of Cu, Ag Ions in the Medium and Culture Time

**DOI:** 10.3390/ijms21124401

**Published:** 2020-06-20

**Authors:** Piotr T. Bednarek, Renata Orłowska

**Affiliations:** Plant Breeding and Acclimatization Institute—National Research Institute, 05–870 Błonie, Radzików, Poland; r.orlowska@ihar.edu.pl

**Keywords:** copper and silver ions, de novo methylation DNA demethylation, sequence variation, time of in vitro culture

## Abstract

During plant tissue cultures the changes affecting regenerants have a broad range of genetic and epigenetic implications. These changes can be seen at the DNA methylation and sequence variation levels. In light of the latest studies, DNA methylation change plays an essential role in determining doubled haploid (DH) regenerants. The present study focuses on exploring the relationship between DNA methylation in CG and CHG contexts, and sequence variation, mediated by microelements (CuSO_4_ and AgNO_3_) supplemented during barley anther incubation on induction medium. To estimate such a relationship, a mediation analysis was used based on the results previously obtained through metAFLP method. Here, an interaction was observed between DNA demethylation in the context of CG and the time of culture. It was also noted that the reduction in DNA methylation was associated with a total decrease in the amount of Cu and Ag ions in the induction medium. Moreover, the total increase in Cu and Ag ions increased sequence variation. The importance of the time of tissue culture in the light of the observed changes resulted from the grouping of regenerants obtained after incubation on the induction medium for 28 days. The present study demonstrated that under a relatively short time of tissue culture (28 days), the multiplication of the Cu^2+^ and Ag^+^ ion concentrations (‘Cu*Ag’) acts as a mediator of demethylation in CG context. Change (increase) in the demethylation in CG sequence results in the decrease of ‘Cu*Ag’, and that change induces sequence variation equal to the value of the indirect effect. Thus, Cu and Ag ions mediate sequence variation. It seems that the observed changes at the level of methylation and DNA sequence may accompany the transition from direct to indirect embryogenesis

## 1. Introduction

Studies on in vitro tissue culture-induced variation (TCIV) revealed that plant regeneration requires cell reprogramming and implicate a broad range of genetic and epigenetic modifications [1]. In this context, DNA methylation (de novo methylation and demethylation) plays a key role for in vitro plant regeneration [2]. Indeed both de novo methylation and demethylation work in concert maintaining homeostasis and may result in the silencing of mobile genome elements, proper shaping of the chromatin structure, or adequate gene expression [3,4]. Changes in DNA methylation may also lead sequence variation under in vitro culture conditions [5,6].

De novo methylation is itself under a well-recognized epigenetic control [7]. It is the outcome of a common substrate S-adenosyl-L-methionine (SAM) reactivity [8,9] that coordinately with the SAM-dependent DNA methyltransferase transfers methyl group on 5′ position of cytosine [10]. In plants, more than 90% of SAM is used for transmethylation, resulting in nucleic acid, protein, lipid, and other metabolite modifications [11].

In the opposite process, DNA demethylation proceeds during DNA replication [12] or is under epigenetic control [13]. Active DNA demethylation requires 5-mC DNA glycosylases [14] and involves DEMETER LIKE 2&3 (DMT2&3) [15]. The glycosylases remove the 5-methylcytosine base by cleavage of the N-glycosidic (base–sugar) bonds resulting in the apurinic or apyrimidinic sites filled by a DNA polymerase and a DNA ligase [16]. The base excision repair (BER) pathway is not the only mechanism of DNA demethylation. The process could be accomplished via the removal of a DNA fragment filled with new nucleotides by nucleotide excision (NER) or mismatch repair (MMR) mechanism. The other mechanisms assume the removal of 5-methylcytosine via its modification due to, e.g., oxidation of the methyl group leading to 5-hydroxymethylcytosine [17], including further modifications [18]. Active DNA demethylation via deamination of 5-mC generates thymine may result in C → T mutations. The other cytosine modifications, e.g., oxidation of 5-mC, may also contribute to sequence variation [19].

Currently different methods are available to evaluate DNA methylation changes. One is the metAFLP (methylation-sensitive Amplified Fragment Length Polymorphism) approach capable of quantifying sequence and DNA methylation changes based on properties of Acc65I and KpnI isoschizomers [20,21]. The other alternative is the MSAP (Methylation Sensitive Amplification Polymorphism) method that utilizes HpaII and MspI properties that allows the quantification of methylation changes [22]. Moreover, new generation sequencing, exploiting the HpaII and MseI isoschizomer in combination with a semi-quantitative MSAP approach (Semi MSAP), could be a method of choice [22]. MethylRAD [23] or MethylSeq [24] are the other alternatives. The metAFLP approach showed its value in our previous studies on in vitro tissue cultures demonstrating that culture stressful conditions may lead to de novo methylation, demethylation, and sequence variation [5,25]. The semi-quantitative MSAP approach was instead utilized to study the role of copper and silver ions on the regeneration of green plants due to the time of barley in vitro anther culture [2].

In this context it must be recalled that the presence of AgNO_3_ in tissue cultures of monocotyledonous species increased embryogenic callus formation [26,27]. It improves the efficiency of shoot induction and regeneration as well as in vitro rooting [28,29,30]. Silver ions prevent callus necrosis [29], modulate organogenesis [31], intensify shoot formation [32], and green plant regeneration [28]. Therefore, in vitro tissue culture may utilize copper and silver ions as ingredients added to the medium to improve the efficiency of androgenesis or somatic embryogenesis. On the other hand, the two ions may be also responsible for stressful conditions under tissue culture plant regeneration procedures depending on concentration and culture time [33,34].

Copper is involved in multiple functions including electron transport chain in the mitochondria and the plastids [35,36]; it participates in the process of photosynthesis and respiration [37,38], detection of ethylene [39], cell wall metabolism [36], protection against oxidative stress [40], and biogenesis of the molybdenum cofactor [41]. It may also contribute to hydroxyl radicals catalyzed by Fe and Cu ions in the Haber–Weiss cycle [35]. The exposure to a sublethal copper concentration affects the activity of enzymes involved in the Krebs cycle [42]. Copper also acts as a cofactor for ethylene binding to the ethylene receptor 1 (ETR1) protein [39].

Silver and copper ions are of comparable sizes. Thus, silver ions may replace Cu ions [43] in many complexes, including mitochondrial complex III [44]. Moreover, Ag^+^ and Cu^2+^ may form complexes with ethylene [45,46] and silver ions may block ethylene action in plants and inhibit its receptors [45]. The ethylene biosynthesis begins with methionine being converted into 1-aminocyclopropane-1-carboxylic acid (ACC) in the presence of SAM and ACC synthetases (Yang cycle). ACC may be a precursor of enzymatic and non-enzymatic copper-stimulated ethylene production [47]. Interestingly, demethylation in the CG context impact on the expression of several genes involved in the ethylene synthesis and signaling pathway [48], indicating a putative regulatory role of DNA methylation [49]. Moreover, copper also participates in a broad range of biochemical pathways that cross with DNA methylation [50].

Although the copper and silver ions may participate or affect many pathways [51], little is known on their input to sequence variation under in vitro tissue culture plant regeneration conditions. Thus, understanding the action of the two ions is crucial for in vitro plant regeneration.

The present study aims at the evaluation of the relationships between DNA methylation affecting CG and CHG change and sequence variation due to the action of copper and silver ions during the anther culture in vitro plant regeneration of barley.

## 2. Results

The in vitro anther tissue cultures of barley were performed under nine distinct conditions (trials M1-M9) varying in the Cu^2+^, Ag^+^ ion concentrations, and the time of in vitro culture (Table 1) allowed the regeneration of 35 plants from a single donor plant. The phenotype (height, leaf width, tillering), of the obtained regenerants being doubled haploids did not differ from the donor plant, which was a source of explants for androgenesis in anther culture.

The metAFLP genotyping allowed the identification of 407 markers shared among the donor plants and regenerants [2]. The metAFLP quantitative characteristics related to demethylation (DMV) of the CG, CHG (H = C, A, and T) sequence contexts, demethylation de novo methylation (DNMV) and sequence variation (SV) were evaluated (Table 1). The time value of in vitro tissue cultures (days) was used as a moderator variable, and the multiplication of the Cu^2+^ and Ag^+^ concentrations ‘Cu*Ag’ as mediator variable (Table 1).

### Moderated Mediation

**Figure 1:**
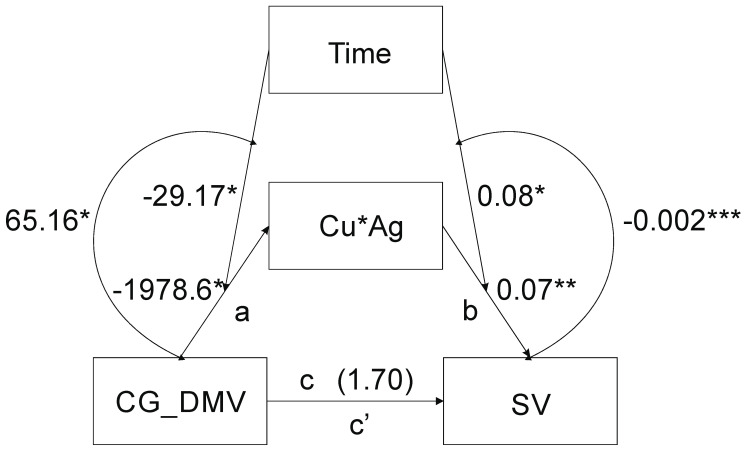
The CG_DMV reflects the CG sequence contexts demethylation; SV states for sequence variation; time indicates the time of in vitro tissue cultures. Curved arrows represent interactions: left side—CG_DMV × time; right side—‘Cu*Ag’ × time. The numbers by the time arrows indicate moderation coefficients; the numbers by the arrows between CG_DMV and ‘Cu*Ag’ (path a), as well as between ‘Cu*Ag’ and SV (path b), reflect unstandardized *B* coefficients of mediation; the number above CG_DMV and SV arrow is the *B* coefficient of direct effect (*DE*) (path c), CG_DMV→’Cu*Ag’→SV reflects indirect effect (path c’). *—*p* < 0.05; ** *p* < 0.001.

Mediation analysis including time of in vitro anther tissue cultures as a moderator of the relationship between CG_DMV and ‘Cu*Ag’ (‘a’) and between ‘Cu*Ag’ and SV (‘b’) paths where CG_DMV was an independent variable, SV was the dependent variable and ‘Cu*Ag’ a mediator shows that the model was significant (*F(3,31)* = 5.21, *p* = 0.005, *R^2^* = 0.33) (Figure 1).

**Figure 2:**
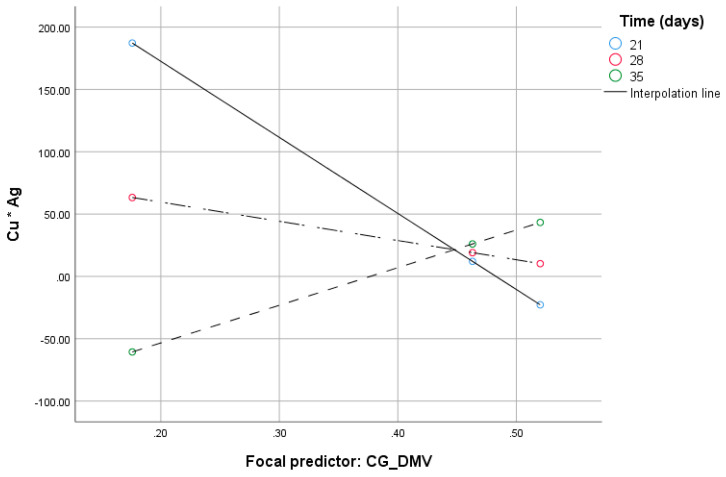
Conditional effect of the CG_DMV (focal predictor) at values of the time of in vitro anther cultures under different ‘Cu*Ag’ values.

The CG_DMV variable (*B* = 1978.6, 95% *CI* [3490.00:467.2]), the time of in vitro cultures (*B* = 29.17, 95% *CI* [55.69:2.64]) and their interaction (*B* = 65.16, 95% *CI* [6.89:123.42]) were significant. The effect of the moderation beyond the main effects (the test of highest order unconditional interaction) *R^2^_chng_* was significant (*F(1.31)* = 5.2, *p* = 0.03) and explained 11.16% of variance due to moderation by the time. With increased CG_DMV, ‘Cu*Ag’ decreases at the lowest value of the time of in vitro tissue cultures (Figure 2). Johnson–Neyman’s statistics indicated that the conditional effect of the CG_DMV (focal predictor) at values of the time (moderator) of in vitro anther cultures was significant within the range of 21–25.9 days of in vitro tissue cultures (not shown).

**Figure 3:**
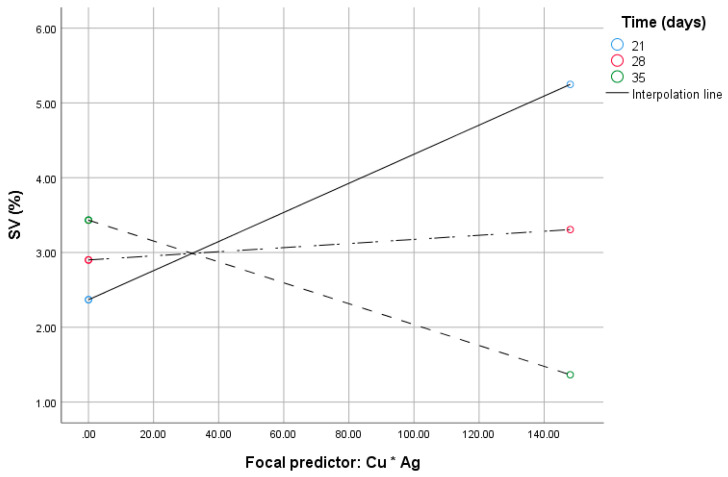
Conditional effect of the ‘Cu*Ag’ (focal predictor) at values of the time of in vitro anther cultures under varying SV.

When the CG_DMV and ‘Cu*Ag’ (the mediator) were considered simultaneously, the model was significant (*F(4.30)* = 101.55, *p* < 0.0001, *R^2^* = 0.9312), whereas path c was not (*B* = 1.7, 95% CI [–0.43:3.44])); however, that of path b was (*B* = 0.07, 95% *CI* [0.056:0.083]). The time of in vitro anther cultures (*B* = 0.08, 95% *CI* [0.012:0.14]), the interaction of the ‘Cu*Ag’ and the time (*B* = –0.002, 95% *CI* [–0.003:–0.0018]) were also significant as indicated by the 95% bootstrap confidence intervals. The highest order unconditional interaction between ‘Cu*Ag’ and the time was significant (*F(1.30)* = 73.91, *p* < 0.001). Up to 16.94% (*R^2^_chang_* = 0.1694) of variance beyond the main effect was due to the moderation by the time. With increased ‘Cu*Ag’ values, SV increases at the lowest value of time for the in vitro tissue cultures (Figure 3). Johnson and Neyman’s statistics indicated that moderation by the time was significant up to 27.7 days of in vitro tissue cultures (not shown).

The interaction between CG_DMV and ‘Cu*Ag’ was significant (*F(1.29)* = 27.51, *p* < 0.0001). The indirect effect (*IE*) of the moderated mediation equaled to *B* = –11.87 (*SE* = 5.31, 95% *CI* [–21.72:–0.17]) and direct effect (*DE*) *B* = 1.7 (*SE* = 0.85, 95% *CI* [–0.04:3.44], respectively. The variance accounted for values of mediated variables accounted for 87.47%. According to the Goodman test the moderated mediation was significant (*Z* = 2.60, *p* < 0.009).

When CHG_DMV, CG_DNMV, and CHG_DNMV were tested as the putative focal predictors of a similar mediation model as described above, both simple mediations and the moderated mediations were not significant (not shown).

**Figure 4:**
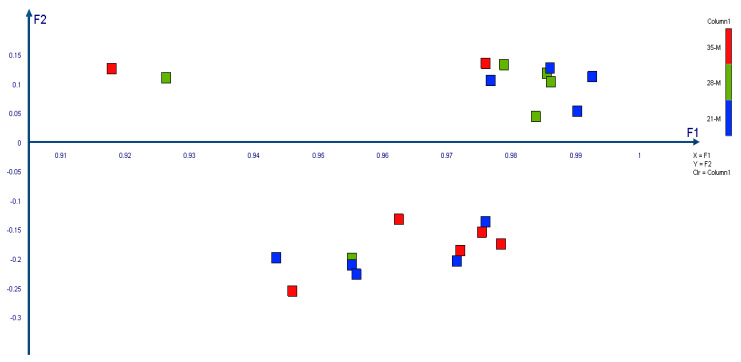
Principal Component Analysis was performed in XLSTAT by Addinsoft 2020.1.1 software (Paris, France). F1 states for the first component whereas F2 for the second one.

Principal Component Analysis based on metAFLP marker data showed that the first component explained 95.2% of the variation, whereas the other 2.1% (Table 2). Cronbach’s alpha equals 0.999. The regenerants formed two groups of data. The first one consists mainly of 28 and 21 days of tissue culture regenerants, whereas the second group of those after 28 and 35 days (Figure 4).

AMOVA demonstrated that the regenerants derived after 21 and 28 days of anther tissue cultures were separated by 0.078, whereas those after 28 and 35 days by 0.164 as indicated by *Φ_PT_* values. Only the later difference was significant (*p* = 0.16) (Table 3).

## 3. Discussion

It is a well-understood fact that DNA methylation changes may contribute to sequence variation under in vitro tissue culture conditions [52]. During DNA demethylation, methylated cytosines may be removed from the DNA, i.e., via base excision repair pathway or 5mC may undergo further modifications [18] also identified by the cell repair system. The respective modifications are usually repaired; however, they may be a source of mutations in some instances. An example here is the 5mC deamination of cytosine. As deamination of C results in T (C → T mutations), such a change is not recognized by the repair system [19]. However, it is not superficial that some elements such as copper and silver ions may participate in sequence variation [53]. Thus, they may also influence DNA under tissue culture conditions. It is not clear whether copper and possibly silver affect G and C as indicated in the case of tobacco cells [54] or maybe specific methylated sequence contexts might be involved. Moreover, the role of time of in vitro tissue cultures in the relationship between DNA methylation and sequence variation under the presence of copper and silver ions is not clear.

The presented moderation mediation analysis demonstrated that, under certain conditions of tissue cultures (Trial M1–M9), demethylation in the CG sequence context might result in sequence variation. The process is mediated by the presence of copper and silver ions added into the in vitro induction medium. Most probably, the pathways that require the presence of copper as a cofactor are affected. The two ions, among others, are involved in glycolysis [55], lipid peroxidation [56,57], and ethylene biosynthesis [46]. The mentioned pathways may contribute to DNA methylation change either via signaling mechanisms or modifying cytosine residues. We tend to think that, under in vitro tissue culture stressful conditions, modified cytosines are not adequately repaired, increasing the level of sequence variation identified among regenerants [20,21]. The presented data reveal that special care is needed utilizing the two ions for in vitro anther cultures as they may influence many biochemical pathways.

Another aspect of the study is the role of time of in vitro anther cultures. Based on our data, the moderation of the time of culture is significant up to 27–28 days. A possible explanation of the role of time is that, after 27 to 28 days of tissue culture, direct embryogenesis proceeding from microspores switches to an indirect one from callus. The development of callus after a particular time of tissue cultures that may serve as a source of tissue for indirect embryogenesis was observed by the others [58] supporting the later notion.

The analysis of the differences among a regenerant derived after 21, 28, and 35 days demonstrates that the 28-day regenerants are distinct at the DNA marker level from those derived after 35 days. Moreover, most of the 28-day-derived regenerants formed a tight group partly mixed with some 21-day regenerants. Such grouping provides evidence that the regenerants were much more similar to each other than in the other cases. AMOVA also supported the results of the principal component analysis, showing that the 35-day regenerants were apart from those derived after 28 days. As the analysis was carried out on the metAFLP markers based on the KpnI/MseI platform (dedicated to recognize sequence changes) the presented data suggest that regenerants derived after 28 days of in vitro tissue culture differ in respect to sequence variation. However, the background of the phenomenon putatively affecting tissue culture within a short time around 28 days of tissue culture is not understood. Thus, specially designed studies are required to investigate the issue. However, we guess that the presented results reflect a switch of tissue culture from direct to indirect embryogenesis.

Interestingly, no evidence of mediation or moderated mediations were evaluated when CHG_DMV or CG_DNMV or CHG_DNMV were used as predictors of mediations. The reason for such results is not apparent; however, CG methylation plays a role in modulating the expression of genes involved in ethylene synthesis and signaling transduction [48]. One option is that the metAFLP approach, due to the relatively low sample size, failed to identify enough variation that could be sensed by moderated mediation analysis in the case of CHG_DMV or CG_DNMV or CHG_DNMV predictors. As the power of statistics was 0.3, we cannot exclude such a situation. If so, the other types of sequence contexts exhibiting DNA methylation change are less prone to sequence variation. Alternatively, it is also possible that CHG sequence contexts methylation change and CG de novo methylation are controlled by distinct mechanisms. It should be stressed that, despite the sample size and low-moderate statistics power, we succeeded in identifying a significant model when CG_DMV was used as a predictor. The significance of the model was confirmed by the Goodman test and bootstrapping of the indirect effects. Bootstrapping is an adequate test, even for small sample sizes ranging from 20 to 80 [59,60]. However, the determination of the sample size is not straightforward for mediation analysis, as there is no simple formula available to carry out this task [61]. Thus, further studies based on an enlarged sample size might be needed to confirm our results.

Remarkably, however, the sequence variation due to CG_DMV requires Cu ions that are involved in many biochemical pathways where copper is a cofactor (i.e., respiratory chain). Changes in the respiratory chain (i.e., due to substitution of copper by silver ions) may affect S-adenosyl L-methionine synthesis, a principal methylation agent in the cell, and a source of radicals that may influence DNA that crosses with DNA repair systems [62]. Thus, the crucial role of copper and silver ions in the model suggests that sequence variation induced within CG sequences affected by demethylation may also involve processes that we could not detect.

## 4. Materials and Methods

Barley (*Hordeum vulgare* L.) cultivar NAD2 provided by Poznan Plant Breeders LTD-Nagradowice (Poland) constituted the material for study. The conditions for obtaining donor plants (D) have been described with all technical details before [21]. From the pool of the 24 donor plants previously obtained, one donor plant and 35 regenerants obtained from it were selected for the presented experiment. Regenerants were derived by androgenesis in anther culture. A detailed description of in vitro cultures has previously been provided [21]. The critical conditions for obtaining regenerants are described below. The cut spikes of donor plants were stored in the dark at 4 °C. Subsequently, the anthers were plated on the N6L induction medium containing macro- and microelements [63] supplemented with 2 mg l^−1^ 2,4-D, 0.5 mg l^−1^ NAA and kinetin. The induction medium was prepared in nine variants (trail M1–M9). Conditions for anther incubation on the induction medium included supplementation with CuSO_4_ and AgNO_3_ salts; individual trials differed in incubation time (Table 1). According to the time regime corresponding to each trial, the anthers were incubated in the dark at 26 °C. Regenerants were regenerated on the K4NB medium [64] supplemented with 0.225 mg l^−1^ BAP at 26 °C and 20µE m^−2^s^−1^ light intensity.

DNA isolation performed from fresh leaves of a donor plant and its regenerants (35 plants) was conducted with the DNeasy MiniPrep kit (Qiagen). The usefulness of DNA for analysis has been verified by spectrophotometric quantity and its electrophoresis integrity in an agarose gel. The metAFLP approach was used for genotyping and quantifying metAFLP characteristics: DNA demethylation (DMV), de novo methylation (DNMV) characteristics within CG and CXG sequence contexts, and sequence variation (SV) characteristics. All metAFLP characteristics were calculated based on AFLP Molecular markers using formulas evaluated on the interpretation of Acc65I and MseI isoschisomer properties. The quantitative description of the metAFLP characteristics was expressed as a percentage. The exact method of calculating individual metAFLP characteristics, including a dedicated spreadsheet (Excel), was previously published [2].

The minimum population size was calculated in G-Power software [65]. Squared multiple correlation *p^2^* was set to 0.1 to calculate effect size *f^2^* at *D* = 0.05 with three variables and power (1-*β* error prob) set to 0.28.

Mediation analysis was performed in SPSS software v. 26 (https://www.ibm.com/support/pages/node/874712) by means of A. F. Hayes Process v. 3.4 macros [66]. Model nr 58 was exploited. The DMV (demethylation), CG_DMV (demethylation of the CG contexts) and CHG_DMV (demethylation of the CHG contexts, where H = C, A, and T) were used as focal predictors, sequence variation was used as a dependent variable, ‘Cu*Ag’ was a mediator whereas the time of in vitro tissue cultures as a moderator.

Principal Component Analysis (PCA) was carried out in XlStat software [67]. Varimax (Kaiser normalization) was applied.

Analysis of molecular variance (AMOVA) was performed in GENALEX (Excel add-in software) [68]. The metAFLP markers (based on KpnI/MseI platform were used for the comparison of anther-derived regenerants after 21, 28 and 35 days of in vitro tissue culture of barley. Probability values based on 999 permutations were evaluated.

## 5. Conclusions

The present study demonstrates that, under a relatively short time of tissue culture (28 days), the multiplication of the Cu^2+^ and Ag^+^ ion concentrations (‘Cu*Ag’) acts as a mediator of CG_DMV. Change (increase) in the CG_DMV is associated to a decrease of ‘Cu*Ag’ and that change induces sequence variation equal to the value of the indirect effect. At least under in vitro conditions tested by us, the role of time is limited to a particular period (28 days). Further experiments are needed to evaluate whether this time is species specific.

## Figures and Tables

**Table 1:** Demethylation of the CG, CHG sequence contexts (DMV), demethylation, de novo methylation (DNMV) expressed in % are independent variables of mediation, whereas sequence variation (SV) frequencies (%) is a dependent variable evaluated in the obtained regenerants, using ‘Cu*Ag’ (multiplication of Cu^2+^ and Ag^+^ ion concentrations) as Mediator variable and time of in vitro culture as Moderator variable. R: regenerant; Trial: reflects varying the concentration of Cu^2+^ and Ag^+^ as well as time of in vitro culture. *M*: Mean; *SD*: standard deviation.

R	Trial	Cu^2+^ (µM)	Ag^+^ (µM)	Independent Variable			Mediator	Dependent Variable	Moderator
				CG_DMV (%)	CHG_DMV (%)	CHG_DNMV (%)	Cu*Ag	SV (%)	Time (Days)
1	M1	0.1	0	0.46	1.16	0.69	0	3.71	21
2	M1	0.1	0	0.23	1.16	0.7	0	2.55	21
3	M1	0.1	0	0.23	1.16	0.7	0	2.55	21
4	M1	0.1	0	0.23	1.16	0.7	0	2.78	21
5	M1	0.1	0	0.46	1.16	0.93	0	3.24	21
6	M2	0.1	10	0.46	1.16	0.7	0	2.55	28
7	M2	0.1	10	0.47	1.16	0.7	0	2.32	28
8	M2	0.1	10	0.46	0.93	0.7	0	2.55	28
9	M3	0.1	60	0.46	0.93	0.7	0	2.55	35
10	M3	0.1	60	0.46	1.16	0.69	0	3.71	35
11	M3	0.1	60	0.46	1.16	0.7	0	3.94	35
12	M3	0.1	60	0.46	0.93	0.7	0	2.55	35
13	M3	0.1	60	0.46	0.92	0.69	0	4.38	35
14	M4	5	60	0.46	0.93	0.7	300	2.55	28
15	M4	5	60	0.46	0.93	0.7	300	2.32	28
16	M5	5	0	0.46	0.93	0.7	0.01	2.55	35
17	M5	5	0	0.46	0.93	0.69	0.01	3.71	35
18	M5	5	0	0.7	0.93	0.93	0.01	3.47	35
19	M5	5	0	0.7	1.16	0.69	0.01	3.71	35
20	M6	5	10	0	0.93	0.7	50	2.55	21
21	M6	5	10	0.46	0.93	0.7	50	2.55	21
22	M6	5	10	0	0.93	0.7	50	2.55	21
23	M6	5	10	0.23	0.93	0.7	50	2.55	21
24	M6	5	10	0.23	0.93	0.7	50	2.32	21
25	M7	10	10	0.69	1.16	0.69	100	4.63	35
26	M7	10	10	0.46	1.16	0.7	100	2.55	35
27	M7	10	10	0.46	0.93	0.69	100	3.94	35
28	M8	10	60	0	0	0.98	600	13.52	21
29	M8	10	60	0	0	0.74	600	13.52	21
30	M8	10	60	0	0	0.74	600	14.01	21
31	M9	10	0	0.93	1.16	0.69	0.01	4.4	28
32	M9	10	0	0.23	0.93	0.7	0.01	2.78	28
33	M9	10	0	0.23	0.93	0.7	0.01	2.78	28
34	M9	10	0	0.23	0.93	0.7	0.01	2.55	28
35	M9	10	0	0.93	1.17	0.7	0.01	3.49	28
*M*		4.75	20.29	0.39	0.94	0.72	84.29	3.95	27.8
*SD*		4.15	25.84	0.24	0.31	0.07	176.05	3.09	5.99

**Table 2:** The arrangement of Principal Component Analysis results performed on metAFLP KpnI/MseI derived markers explaining the variation among regenerants. F1 and F2 stat for the first and second component, respectively.

	F1	F2
Eigenvalue	33.322	0.755
Variability (%)	95.204	2.158
Cumulative (%)	95.204	97.362

**Table 3:** Pairwise *Φ_PT_* values (below diagonal). Probability values based on 999 permutations are shown above diagonal. 21-M, 28-M and 35-M reflect regenerants derived after 21, 28 and 35 days of in vitro tissue culture, respectively.

*Φ*_PT_	21-M	28-M	35-M
21-M	0.000	0.105	0.345
28-M	0.072	0.000	0.016
35-M	0.000	0.164	0.000

## Abbreviations

2,4-D	2,4-dichlorophenoxyacetic acid
AMOVA	Analysis of Molecular Variance
ASS	1-aminocyclopropane-1-carboxylic acid
BAP	6-benzylaminopurine
BER	base excision repair
D	donor plants
DH	doubled haploid
DMT2&3	DEMETER LIKE 2&3,
DMV	demethylation
DNMV	de novo methylation
ETR1	ethylene receptor 1
metAFLP	methylation sensitive Amplified Fragment Length Polymorphism
MMR	mismatch repair
MSAP	Methylation Sensitive Amplification Polymorphism
NAA	napthaleneacetic acid
NER	nucleotide excision
PCA	Principal Component Analysis
SAM	S-adenosyl-L-methionine,
